# Feel your stride and find your preferred running speed

**DOI:** 10.1242/bio.014886

**Published:** 2015-12-23

**Authors:** Thibault Lussiana, Cyrille Gindre

**Affiliations:** 1 Research Unit EA4660, Culture Sport Health Society and Exercise Performance Health Innovation Platform, Bourgogne Franche-Comte University, Besançon 25000, France; 2 Volodalen Company, Research and Development Department, Chavéria 39270, France; 3 Volodalen Suisse Company, Research and Development Department, Leysin 1854, Switzerland

**Keywords:** Running pattern, Self-selected speed, Pleasure, Biomechanics, Adaptation

## Abstract

There is considerable inter-individual variability in self-selected intensity or running speed. Metabolic cost per distance has been recognized as a determinant of this personal choice. As biomechanical parameters have been connected to metabolic cost, and as different running patterns exist, we can question their possible determinant roles in self-selected speed. We examined the self-selected speed of 15 terrestrial and 16 aerial runners, with comparable characteristics, on a 400 m track and assessed biomechanical parameters and ratings of pleasure/displeasure. The results revealed that aerial runners choose greater speeds associated with shorter contact time, longer flight time, and higher leg stiffness than terrestrial runners. Pleasure was negatively correlated with contact time and positively with leg stiffness in aerial runners and was negatively correlated with flight time in terrestrial runners. We propose the existence of an optimization system allowing the connection of running patterns at running speeds, and feelings of pleasure or displeasure.

## Abstract (precis)

**Summary:** Aerial and terrestrial human running patterns self-select different pleasure running speeds. We suggest the existence of an optimization system allowing association of runners' stride with running speed.

## INTRODUCTION

There is a speed at which every individual prefers to run. Thus, considerable inter-individual variability exists in self-selected levels of exercise intensity. In a study by Lind et al. (2005), although the peak of the self-selected intensity distribution was centred near 100% of oxygen consumption at the ventilatory threshold, individual values ranged from 62% to 160% at the end of a 20-min exercise period. The determinants of self-selected exercise intensity have been investigated by mainly three types of studies. Subjects' characteristics, such as age, aerobic capacity, and body mass and composition could play a role (Ekkekakis et al., 2011). The affective responses (i.e. pleasure-displeasure) with the use of the maximization of pleasure and/or the minimization of displeasure as guides could serve as regulators (Ekkekakis et al., 2011). Finally, the need to maximize the distance travelled by minimizing the metabolic cost could guide the exercise intensity choice (Alexander, 2002). For this, individuals choose stride length and duty factor, i.e. contact (*t*_c_) and flight (*t*_f_) times relation, to make the running form economic (Alexander, 2002). In connection with that last point, we can raise the question of a possible role of biomechanical parameters in the choice of exercise intensity or running speed.

Recently, Gindre et al. (2015) showed that runners could be divided into two global running patterns, i.e. aerial (AER) and terrestrial (TER). At standardised speeds, the two groups elicited different biomechanical parameters leading to different strategies to optimize running economy (Gindre et al., 2015). AER was associated with shorter *t*_c_, longer *t*_f_, and greater leg stiffness (*k*_leg_) than TER (Gindre et al., 2015). Based on the biomechanical analysis, the author hypothesised that AER relies on the stretch-shortening cycle and the return of elastic energy to minimize energy expenditure, whereas the TER minimizes energy expenditure through reduced vertical oscillation and external work (Gindre et al., 2015). On the speed continuum, the biomechanical parameters associated with AER correspond well to the higher speed-induced parameters, while those associated with TER match the lower speed-induced parameters (Padulo et al., 2012).

Therefore, the aim of this study was to verify the hypothesis of a biomechanical role on self-selected speed by comparing speed chosen in the AER and TER groups. As AER demonstrates kinematic parameters that are habitually associated with high speed, we hypothesized that AER self-selected a greater running speed than TER to match to their preferences.

## RESULTS

**Fig. 1.**
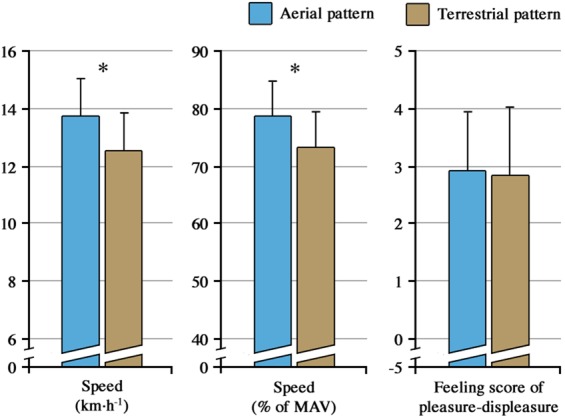
**Absolute (km h^−1^) and relative [% of maximal aerobic velocity (MAV)] self-selected speeds, and feeling score of pleasure-displeasure in aerial and terrestrial patterns.** Feeling scores of pleasure-displeasure recorded after the 15-min run were similar between the two groups, while absolute and relative self-selected speeds were greater in the aerial pattern than in the terrestrial pattern. *P<0.05: significant difference between aerial and terrestrial groups. Data presented as mean±s.d.

AER runners chose a greater speed [expressed in km h^−1^ or in % of maximal aerobic velocity (MAV)] than TER runners at similar ratings on the pleasure-displeasure scale (Fig. 1). At self-selected speeds, AER and TER exhibited specific biomechanical parameters (AER vs TER, *t*_c_: 255±25 vs 284±27 ms; *P*=0.003, *t*_f_ : 101±24 vs 80±31 ms; *P*=0.026, and *k*_leg_: 9.8±1.1 vs 8.9±1.1 kN m^−1^; *P*=0.037) in line with previously reported results at a standardised speed (Gindre et al., 2015). Interestingly, significant relationships were found between the score of pleasure-displeasure for *t*_c_ [*r*=−0.60 (−0.82; −0.23); *P*=0.015] and for *k*_leg_ [*r*=0.49 (0.08; 0.76); *P*=0.054] in the AER group. In the TER group, a large correlation between feeling score and *t*_f_ [*r*=−0.58 (−0.81; −0.18); *P*=0.024] was observed. It is of note that there are only unclear relationships for *t*_c_ [*r*=0.22 (−0.24; 0.61); *P*=0.463] and *k*_leg_ [*r*=0.19 (−0.27; 0.59); *P*=0.511] in TER and for *t*_f_ [*r*=0.24 (−0.21; 0.60); *P*=0.361] in AER.

## DISCUSSION

**Fig. 2.**
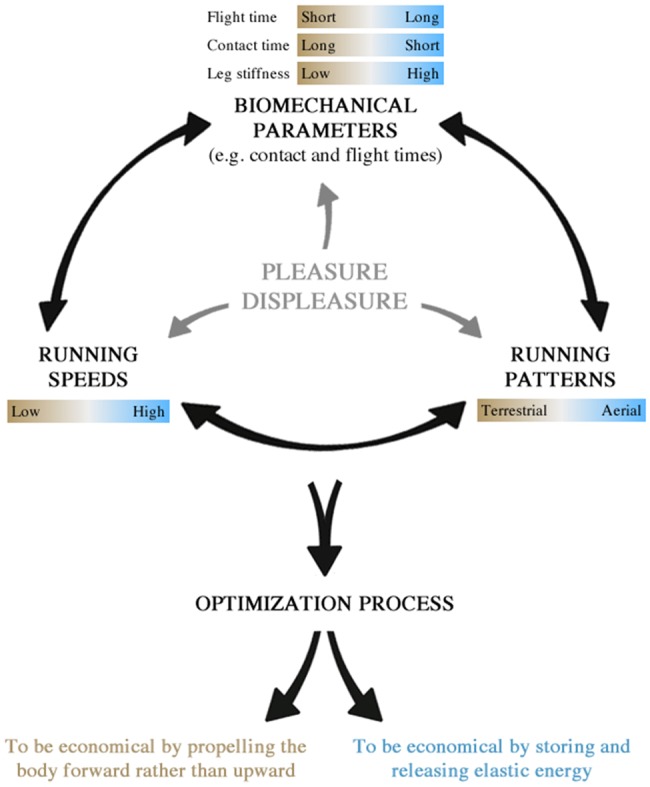
**Schematic representation of the interactions within the three-party system.** The system is based on three elements including running patterns, running speeds, and biomechanical parameters. Each element can evolve through continuums and lead to different optimization strategies. Finally, the strategies seem to be mediated by the feeling of pleasure-displeasure.

For the first time, this study established links between running patterns, running speeds and biomechanical parameters. We propose the existence of a three-party system based on these elements, which can evolve through continuums presented in Fig. 2. Within this system, we hypothesize that two strategies of optimization exist. The first strategy is used by TER runners at low speed and relies on the ability to generate forces over a longer period of time (Kram and Taylor, 1990), i.e. long *t*_c_, and low vertical displacements (Hébert-Losier et al., 2015), i.e. short *t*_f_. The second strategy is used by AER runners at higher speed and relies on the ability to use the stretch-shortening cycle (Dumke et al., 2010), i.e. short *t*_c_ and high *k*_leg_. The first strategy refers to the ability to propel the body forward rather than upward, while the second refers to the ability to store and release elastic energy. Moreover, in view of the correlations between biomechanical parameters and the feeling scores, these two strategies seem to be at least partly mediated to the feeling of pleasure-displeasure. We therefore suggest that individuals increase their ratings of pleasure-displeasure in adapting to external variables, e.g. running speed, in which they perceive to be biomechanically efficient.

This optimisation system is consistent with animal studies. It has been shown that (1) stride kinematics (e.g. contact time) can be considered as a collective result of the intrinsic properties (e.g. control strategies) of the locomotors system (e.g. ground-dwelling) (Zaaf et al., 2001), (2) variation in whole-body posture (e.g. centre of mass position) may reflect different environments (e.g. climbing habitat) (Clemente et al., 2013), and (3) habituation to a specific environment (e.g. climbing habitat) induces biomechanical adaptations (e.g. stride frequency) (Clemente et al., 2013). In our study, these three points are translated in the following way: (1) biomechanical parameters can be considered as results of the rebound and forward propulsion abilities, (2) running patterns may favour different running speeds, and (3) running speed induces different biomechanical adaptations. In fact, the biomechanical differences between AER and TER could translate to the two principles of energy economy associated with different running speeds in the same way as kinematic differences among species of lizard can mask higher-level performance traits typically associated with environment variation (Clemente et al., 2013). This suggestion is consistent with the concept of niche partitioning, which is based on the understanding that a species can occupy a particular ecological microhabitat by having a behaviour adapted to suit that environment (MacArthur and Levins, 1967).

The choice of a preferred speed could be affected by the performance level and the intensity of the training habits (Zamparo et al., 2001). It is reasonable to expect that runners with a higher performance level train at higher intensities and therefore self-select a higher running speed for pleasure. However, no differences in performance levels (MAV) or usual training intensities at baseline were observed between our AER and TER runners (Table 1). In the same way, age has been shown to influence self-selected speed and running biomechanics, and might have confounded our results. Indeed, speed chosen, vertical oscillation of the centre of mass, and flight time are reduced with maturation (Cavagna et al., 2008; Ekkekakis et al., 2011) implying a more TER classification in older participants. However, no difference in the mean age of TER and AER runners was observed in this study. More in-depth investigations of the effect of age, performance level, and training habits are warranted to further understand their effects on Volodalen^®^ classification. In this classification method, runners with a subjective score in the middle of the scale (*n*=3, *V*^®^_score_=15) have been arbitrarily associated with the terrestrial pattern. We could expect that these individuals relied on both of the described optimization systems to a certain extent. For ease-of-use of the scale and to simplify understanding, we have chosen to divide the running pattern of individuals into two categories only. A future study focused on the validity of the Volodalen^®^ method and appropriateness of classifying runners into two (i.e. *V*^®^_score_ TER≤15 and AER>15) versus three categories (i.e. *V*^®^_score_ TER≤11, MIX 12 to 18, and AER≥19) could assist in answering this question.

**Table 1.**
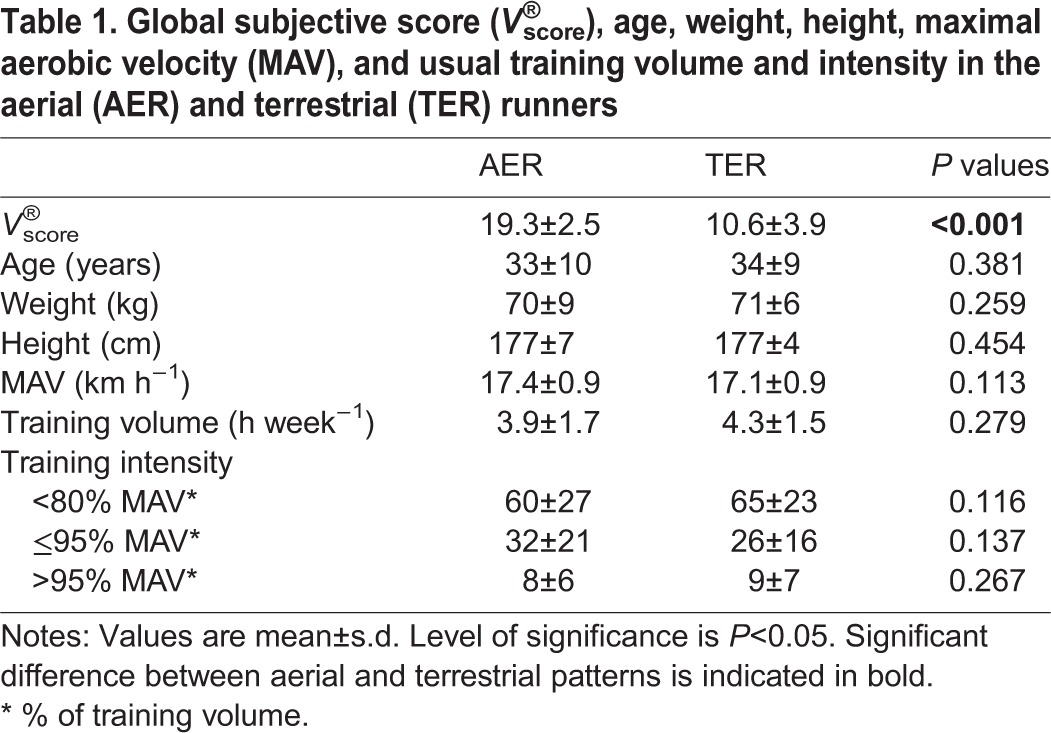
**Global subjective score (*V*^®^_score_), age, weight, height, maximal aerobic velocity (MAV), and usual training volume and intensity in the aerial (AER) and terrestrial (TER) runners**

The present study revealed that AER and TER runners self-selected different running speeds with specific biomechanical characteristics, i.e. longer *t*_c_, shorter *t*_f_, and higher *k*_leg_ in TER compared to AER. We propose the existence of a three-party system referring to different strategies of optimization based on the rebound and the forward propulsion abilities. Moreover, the correlations established between biomechanical parameters correlated to the feeling score (i.e. for *t*_f_ in TER and for *t*_c_ and *k*_leg_ in AER) suggest that the feeling of pleasure-displeasure acts as a magnet and must be taken into account in the global optimized system.

## MATERIALS AND METHODS

### Participants and experimental procedure

Thirty-one well-trained runners (age: 33.1±9.4 years, height: 177.3±5.9 cm, and body mass: 70.5±7.5 kg) participated in this study. MAV of all participants was 17.3±1.0 km h^−1^ as determined using an incremental track test, starting at 10 km h^−1^ and with an increased speed of 0.5 km h^−1^ every minute until exhaustion, one week before data collection (Léger and Boucher, 1980). The university's institutional review board approved the study protocol prior to participant recruitment (CPP: 2014-A00336-41), which was conducted in accordance with the latest amendments of the Declaration of Helsinki.

After a 10-min running warm-up (velocity between 2.5 and 3.5 m s^−1^), participants performed a 15-min running trial at self-selected speeds on an athletic track. Participants were asked to run at their preferred speeds, i.e. where they feel the best and take the most pleasure. No other instruction was assigned to not impair spontaneous behaviour. Each participant ran alone on the track.

### Pleasure-displeasure assessment

The Feeling Scale, consisting of an 11-point single-item scale ranging from +5 (very good) to −5 (very bad), was used to quantify pleasure and displeasure (Hardy and Rejeski, 1989). Participants were asked to report their feeling score of pleasure-displeasure immediately at the end of the 15-min running at self-selected speeds.

### Subjective assessment of running gait

**Fig. 3.**
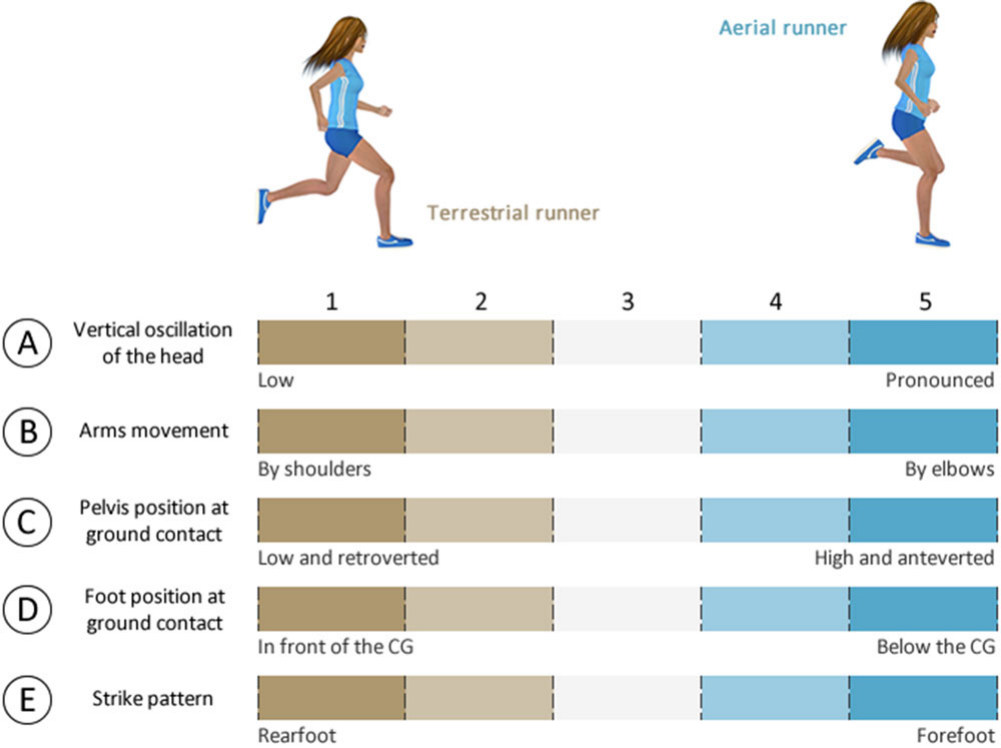
**Subjective grid of the Volodalen^®^ method.** This scale allows the coach to assess the individual running pattern and classifies runners into two categories: aerial and terrestrial patterns.

During the 10-min warm-up, a running coach with more than 10 years of experience using the Volodalen^®^ method (Gindre et al., 2015) focused on the overall movement patterns of participants. The coach graded runners on a 5-item scale (Fig. 3) which ultimately allows the classification of runners based on a global subjective score (*V*^®^_score_) into two different categories: TER (*V*^®^_score_≤15) or AER (*V*^®^_score_>15) groups. The classification procedure demonstrates adequate intra and inter-rater reliability [respectively, coefficient of variation (c.v.): 6.1±7.0% and 6.6±6.5%, paired *t*-test: *P*=0.927 and 0.250], and is fully described elsewhere (Gindre et al., 2015).

In this study, participants' characteristics, running performance and the usual training volume and intensity of AER (*N*=16) and TER (*N*=15) did not show significant differences (Table 1), which allowed us to separate the effect of running patterns on self-selected speed, feeling score of pleasure-displeasure and biomechanical parameters.

### Objective assessment of running gait

Twenty meters of an optical measurement system (Optojump, MicroGate Timing and Sport, Italy) sampling at 1000 Hz was used to record *t*_c_ and *t*_f_ every 400 m. *k*_leg_ was then estimated using a sine-wave model (Morin et al., 2005), as the ratio between the maximal vertical force (*F*_max_) and the maximal leg length deformation, i.e. leg spring compression (*Δ*_L_) calculated with the centre of mass displacement (*Δ*_z_), using the following equations:
(1)
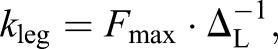

(2)
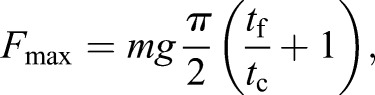

(3)
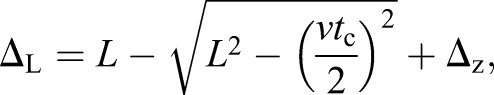

(4)
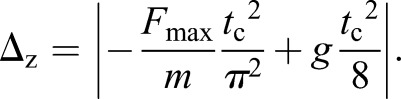


### Statistical analysis

Descriptive statistics are presented using mean±s.d. values and correlation statistics using mean±90% confidence limits (c.l.). Student *t*-tests were used to compare participants' characteristics, self-selected speed, pleasure-displeasure, and biomechanical parameters (mean values from all laps) between AER and TER. Correlation coefficients were used to assess whether the feeling score of pleasure-displeasure correlated with the self-selected speed and biomechanical parameters for AER and TER separately. The following criteria were adopted to interpret the magnitude of the correlation between the different measures: ≤0.1, trivial; >0.1-0.3, small; >0.3-0.5, moderate; >0.5-0.7, large; >0.7-0.9, very large; and >0.9-1.0, almost perfect (Hopkins et al., 2009). If the 90% c.l. overlapped positive and negative values, the magnitude of the correlation was deemed unclear. Statistical significance was accepted when the overall *P* value was <0.05 and was performed using SigmaStat12 (Systat Software Inc., USA) and Hopkins spreadsheets (http://www.sportsci.org).
